# High‐intensity resistance training and collagen supplementation improve patellar tendon adaptations in professional female soccer athletes

**DOI:** 10.1113/EP092106

**Published:** 2024-08-29

**Authors:** Joonsung Lee, David C. Robshaw, Robert M. Erskine

**Affiliations:** ^1^ School of Sport and Exercise Sciences Liverpool John Moores University Liverpool UK; ^2^ Institute of Sport, Exercise and Health University College London London UK

**Keywords:** connective tissue, strength exercise, tendon stiffness, women's football, Young's modulus

## Abstract

We investigated whether 10 weeks of pre‐season soccer training (including high‐intensity resistance exercise) with hydrolysed collagen (COL) supplementation would confer greater changes in patellar tendon (PT) mechanical and material properties compared with placebo (PLA) in professional female soccer athletes. Eleven athletes from the first team squad of a Football Association Women's Championship soccer club volunteered to participate in this study (age, 25.7 ± 4.2 years; height, 1.68 ± 0.04 m; mass, 64.0 ± 4.6 kg). Participants were pair‐matched for baseline knee extensor maximum isometric voluntary contraction torque, age, height and mass and were randomly assigned to the COL group (*n *= 6) or PLA group (*n *= 5). Participants were given 30 g COL or energy‐matched (36.5 g maltodextrin and 8.4 g fructose) PLA, plus 500 mg vitamin C before each training session, which consisted of high‐intensity lower‐limb resistance exercise, plyometric or pitch‐based exercise 3 days/week for 10 weeks during the pre‐season period. We assessed knee extensor maximum isometric voluntary contraction torque and PT properties using isokinetic dynamometry and ultrasonography before and after the intervention. The PT stiffness [COL, +15.4% ± 3.1% (*d *= 0.81) vs. PLA, +4.6% ± 3.0% (*d *= 0.32), *P *= 0.002] and Young's modulus [COL, +14.2% ± 4.0% (*d *= 0.65) vs. PLA, +3.4% ± 2.8% (*d *= 0.15), *P *= 0.004] increased more in COL than in PLA. There was a main effect of training on PT cross‐sectional area (*P *= 0.027), but no interaction effect (*P *= 0.934). To conclude, 10 weeks of pre‐season soccer training (incorporating high‐intensity resistance exercise) with 30 g COL increased PT stiffness and Young's modulus more than training alone in professional female soccer athletes. This has positive implications for improving athletic performance and mitigating injury risk.

## Abstract (highlights)

- **What is the central question of this study?**
  Does 10 weeks of soccer training (incorporating high‐intensity resistance exercise), supplemented with 30 g hydrolysed collagen ingestion, change the mechanical and material properties of the patellar tendon more than training alone in professional female soccer athletes?
- **What is the main finding and its importance?**
  Changes in patellar tendon stiffness and Young's modulus were greater following 10 weeks of training with 30 g collagen supplementation compared with training alone. Given that a stiffer tendon can tolerate greater stress and transmit force more efficiently, combining collagen ingestion with resistance training might help to mitigate injury risk and improve physical performance in this under‐researched population.

## 1 INTRODUCTION

Chronic resistance exercise (RE), i.e., resistance training (RT) (Kongsgaard et al., 2007; Reeves et al., 2003; Seynnes et al., 2009), and chronic plyometric exercise (Foure et al., 2010) are known to improve the morphological and mechanical properties of human tendon. Also, when tendon is exposed to habitual loading, its stiffness and thickness are increased (Couppé et al., 2008, 2021). A stiffer tendon is associated with a faster rate of torque development (Bojsen‐Møller et al., 2005), which is also linked to greater lower‐limb strength and power output (Bojsen‐Møller et al., 2005; Wdowski et al., 2023). Furthermore, given that there is a linear relationship between the Young's modulus of a tendon and its ultimate stress (LaCroix et al., 2013), a stiffer tendon has a greater loading capacity, which might help to reduce tendon injury risk during periods of high mechanical load, such as during soccer training/match play. This is particularly important for female soccer athletes, who demonstrate a higher incidence of soft tissue injuries than their male counterparts (Larruskain et al., 2018). A major injury risk factor in women's soccer is muscle weakness (Crossley et al., 2020), and the most effective method to increase muscular strength and therefore reduce injury risk is to conduct RT. Thus, RT could have multiple benefits for female soccer athletes.

The aforementioned tendon adaptations to RT are likely to be attributable to repeated increases in collagen synthesis known to occur after a single bout of RE (Lee, Tang, et al., 2023b, 2025; Miller et al., 2005). Moreover, collagen synthesis after high‐intensity RE is augmented when supplemented with hydrolysed collagen (COL) ingestion in a dose–response manner, i.e., 30 g > 15 and 0 g hydrolysed collagen (Lee, Tang, et al., 2023). However, only one study to date has investigated the chronic effects of RT supplemented with COL on tendon properties in female athletes (Lee, Bridge, et al., 2023), and the results demonstrated greater increases in tendon stiffness and Young's modulus compared with the placebo (PLA) group. Nevertheless, neither the COL nor the PLA group showed any tendon hypertrophy following the 10 week intervention, which was probably attributable to the relatively low intensity of the bodyweight RE (Lee, Bridge, et al., 2023), because high‐intensity RE is thought to be necessary to induce tendon hypertrophy (Kongsgaard et al., 2007; Seynnes et al., 2009). Furthermore, the participants were academy athletes, hence the volume and intensity of training and match play would probably have been lower than for professional athletes, particularly given that the study by Lee, Bridge, et al. (2023) was performed during the competitive season, when it is more challenging to incorporate high‐intensity RT into soccer training (McQuilliam et al., 2022). Conversely, owing to the low frequency of competitive matches during the pre‐season period, there is greater scope to incorporate high‐intensity RT into training, thus potentially enabling greater tendon adaptation to RT with COL supplementation, although this remains to be investigated.

The primary aim of this study was therefore to investigate the effect of 10 weeks of pre‐season soccer training (incorporating high‐intensity resistance exercise) with 30 g COL supplementation on changes in patellar tendon (PT) properties in professional female soccer athletes. Given that previous studies have found that RT with COL supplementation increased fat‐free mass (Kirmse et al., 2019) and muscle size (Balshaw et al., 2023) more than RT alone, a second aim was to investigate the effect of soccer training and COL on changes in quadriceps muscle thickness. We hypothesized that 30 g COL ingested immediately before each training session (three times per week) would improve morphological and mechanical properties of the patellar tendon more than soccer training alone. Owing to the relatively low composition of essential amino acids [crucial for an optimal muscle protein synthesis response (Tang & Phillips, 2009)] in hydrolysed collagen (Gauza‐Włodarczyk et al., 2017), we hypothesized that COL would not affect the muscle hypertrophic response to RT.

## 2 MATERIALS AND METHODS

### 2.1 Experimental design

This was a single‐blind, single‐centre, randomized controlled trial. Given that the study took place during the coronavirus disease 2019 (COVID‐19) pandemic, which limited personnel permitted on the University and soccer training grounds, the lead researcher was unblinded to participant group allocation (COL or PLA) to ensure that participants were administered the correct supplements. Crucially, however, all participants and the researcher responsible for supervising all training sessions were blinded to group allocation. All participants attended the laboratory for muscle–tendon assessments before and after 10 weeks of soccer training, which took place during the 2021–2022 pre‐season period (July–September 2021) and consisted of a combination of high‐intensity RT, plyometric and pitch‐based soccer training. All muscle–tendon measurements were performed on the right leg in the following order: PT cross‐sectional area (CSA), maximal isometric and isokinetic strength of the knee extensors and flexors (antagonist muscle activation was measured using surface EMG) and PT elongation were measured via a combination of ultrasonography and isokinetic dynamometry. All tests took place between 09.00 and 17.00 h, and the pre/post‐training tests were performed at the same time of day for each participant to avoid potential diurnal effects on intra‐individual pre/post‐training changes (Onambele‐Pearson & Pearson, 2007). Furthermore, participants were instructed not to participate in strenuous physical activity and not to consume alcohol or caffeine in the 24 h prior to testing. Following the baseline assessments, participants were then randomly assigned to one of two groups (COL and PLA) and instructed to ingest their respective supplement three times a week with training for the 10‐week period. Each participant completed all 30 training sessions and ingested all of their 30 respective supplements. Post‐training assessments were performed within 3–5 days after the final supplemented training session.

### 2.2 Participants

A minimal sample size was estimated prior to conducting the study with G*Power software (v.3.1.9.6, Heinrich‐Heine‐Universität Düsseldorf, Düsseldorf, Germany) using the effect size (η_p_^2^ = 0.264) from the time (pre and post 10 weeks of soccer training) × group (COL vs. PLA) interaction (*P* = 0.035) concerning the change in PT Young's modulus (Lee, Bridge, et al., 2023). A minimum of 14 participants was considered necessary to detect an effect of COL versus PLA (two‐way ANOVA; α = 0.05; power = 0.80). To account for an expected 10%–20% participant withdrawal, 18 professional female soccer athletes were recruited from the first team squad of a Football Association Women's Championship soccer club. All volunteers provided written informed consent to take part in this study, which was approved by Liverpool John Moores University Research Ethics Committee (approval number: 19/SPS/054) and complied with the *Declaration of Helsinki*. The study was also registered at https://clinicaltrials.gov/ (identifier: NCT06372080). Participant recruitment began in June 2021, and data collection was completed in August 2021 (a CONSORT flow diagram for this study is shown in Figure S1). Prior to baseline testing and randomized group allocation, three participants became infected with COVID‐19 and had to withdraw from the study. Thus, 15 participants were randomly allocated into COL (*n *= 8) or PLA (*n *= 7) based on pair‐matched age, height, body mass and isometric knee extension (KE) maximal voluntary contraction (MVC) torque. However, four more participants had to withdraw during the course of the study owing to soccer‐related injuries (*n *= 3) or personal reasons (*n *= 1). Therefore, the following participants completed the study: COL (*n *= 6; age, 24.3 ± 3.0 years; height, 1.68 ± 0.03 m; body mass, 64.1 ± 4.8 kg; isometric KE MVC, 190 ± 32N m) and PLA (*n *= 5; age, 27.4 ± 5.1 years; height, 1.69 ± 0.06 m; body mass, 64.1 ± 5.6 kg; isometric KE MVC, 204 ± 37N m). The COL group consisted of two defenders, three midfielders and one forward, and the PLA group consisted of one goalkeeper, two defenders, one midfielder and one forward. Two participants in COL were using an intrauterine device and had been doing so for 3.8 ± 4.5 years, and another participant in the COL group was using an oral contractive pill (Gedarel® 30/150) and had been doing so for 6 years. Three participants in the PLA group were using an oral contractive pill (Rigevidon®, Lucette® and Microgynon® 30) and had been doing so for 7.3 ± 4.2 years. The remaining five participants were ‘normally’ menstruating women, as determined by responses to the ‘low energy availability in females questionnaire’, which was used to screen for menstrual function and use of hormonal contraceptives (Melin et al., 2014). Exclusion criteria for all participants included: a history of lower limb muscle/tendon injuries in the 6 months prior to the start of the study; consumption of nutritional supplementation that purportedly affects muscle–tendon adaptation or recovery (e.g., protein, vitamin C, collagen); being vegan or vegetarian (owing to the mammalian source of collagen); previous anterior cruciate ligament injury where the patellar tendon was used as a graft; and age <18 years or >39 years.

### 2.3 Training and nutritional intervention period

Participants performed four training sessions (Tuesday, Wednesday, Friday and Saturday) and one friendly match (Sunday) per week, which was part of the athletes’ regular training and competition during the pre‐season period. The nutritional supplementation was consumed on three of those sessions every week for 10 weeks consecutively. A typical microcycle with nutritional supplementation was Tuesday (pitch‐based session followed by externally loaded upper‐body resistance exercises), Wednesday (pitch‐based session followed by externally loaded, high‐intensity, lower‐limb resistance exercises) and Friday (externally loaded lower‐limb plyometric exercises followed by pitch‐based sessions). An additional pitch‐based session was conducted on Saturday, which was used as a supplementation day only if participants missed one of their regular supplementation days or the match day. At the beginning of pre‐season, three‐repetition maximum testing was performed for the split squat exercise and rear‐foot elevated split squat, to predict one‐repetition maximum for each athlete's training load for these exercises. In addition to the split squat and rear‐elevated split squat exercises, the externally loaded lower‐limb RE consisted of bilateral ballistic exercises, hip dominant posterior chain exercises and unilateral plantar flexor exercises. Furthermore, Copenhagen adduction and Nordic hamstring exercises were also included in the programme. During the 10‐week training period, RE volume was increased progressively on a weekly basis. Detailed training programmes for the externally loaded lower‐limb RE and plyometric exercises are presented in Table S1.

### 2.4 Nutritional supplementation

Owing to the high standard of athletes participating in this study, all supplements needed to be ‘Informed Sport’ certified as having been tested by the laboratory of the government chemist (LGC) Group anti‐doping laboratory for contamination with banned substances. Participants in the COL group received 90 mL ‘Collagen Liquid’ (GBR Nutrition, UK), which contained 30 g collagen hydrolysate and contained 180 kcal. Participants in the PLA group received 49.3 g ‘Tropical’ flavour ‘GO Electrolyte’ (Science in Sport, UK), which contained 36.5 g maltodextrin and 8.4 g fructose and contained 180 kcal. Each supplement was mixed with water to create a total volume of 250 mL, and all participants were given a 500 mg vitamin C tablet (Elite Vitamin C, Healthspan, UK) to consume immediately after consuming the drink. Participants consumed their supplements in entirety immediately before each training session. All drinks were provided in opaque bottles and, together with the taste‐matching and equal volume of drink, this ensured that participants remained blinded to their allocated group for the entirety of the study. The number of nutritional supplements that participants consumed during the different types of training session is shown in Table S2.

### 2.5 Habitual dietary intake and anthropometry

The height (SECA, model‐217, Germany) and body mass (SECA, model‐875, Germany) of participants were measured to the nearest 0.1 cm and 0.1 kg, respectively. Participants were asked to record their habitual dietary behaviour using a food and drink diary for 3 days (from Thursday to Saturday) during the baseline testing period. This aspect of the study was completed by *n *= 10 (Table 1). Records were analysed with Nutritics professional dietary analysis software (v.5.09, Nutritics, Ireland) to obtain total energy, macro‐ and micronutrient composition. All daily nutritional composition data were presented as absolute values and relative to body mass. ‘Total intake’ was calculated as the sum of habitual intake and nutritional supplementation used in this study.

**TABLE 1:** Energy, macronutrient and micronutrient intake during the pretraining assessment period. Data are means ± SD.

Nutritional composition	COL (*n* = 5)	PLA (*n* = 5)	*t*‐test, *P*‐value
Energy intake			
Habitual intake (kcal day^−1^)	1743 ± 242	1584 ± 328	0.408
Total intake (kcal day^−1^)	1820 ± 242	1661 ± 328	0.408
Carbohydrate intake			
Habitual intake (g day^−1^)	213 ± 31	192 ± 32	0.336
Habitual intake (g kg^−1^ day^−1^)	3.4 ± 0.4	3.0 ± 0.4	0.112
Total intake (g day^−1^)	213 ± 31	212 ± 32	0.956
Total intake (g kg^−1^ day^−1^)	3.4 ± 0.4	3.3 ± 0.4	0.592
Protein intake			
Habitual intake (g day^−1^)	103 ± 39	83.1 ± 25.9	0.367
Habitual intake (g kg^−1^ day^−1^)	1.6 ± 0.5	1.3 ± 0.3	0.239
Total intake (g day^−1^)	116 ± 39	83 ± 26	0.155
Total intake (g kg^−1^ day^−1^)	1.8 ± 0.5	1.3 ± 0.3	0.083
Fat intake			
Habitual intake (g day^−1^)	51.9 ± 13.5	49.8 ± 14.7	0.818
Habitual intake (g kg^−1^ day^−1^)	0.8 ± 0.2	0.8 ± 0.2	0.547
Vitamin C intake			
Habitual intake (mg day^−1^)	82.5 ± 64.0	125 ± 31	0.218
Habitual intake (mg kg^−1^ day^−1^)	1.4 ± 1.1	2.0 ± 0.5	0.279
Total intake (mg day^−1^)	297 ± 64	339 ± 31	0.218
Total intake (mg kg^−1^ day^−1^)	4.8 ± 1.2	5.3 ± 0.5	0.384

### 2.6 Knee extensor and flexor maximal voluntary contraction

Isometric and concentric knee extension (KE) and isometric knee flexion (KF) maximal voluntary contractions (MVCs) were measured on an isokinetic dynamometer (Humac Norm, Computer Sports Medicine, USA). Knee and hip joint angles were set at 90° (0° = full knee extension) and 85° (180° = supine), respectively, and movement was restricted with the use of inextensible waist, chest and thigh straps. The torque signal was interfaced with an analog‐to‐digital converter (MP150, Biopac Systems, USA), sampled at 2 kHz with a personal computer using data acquisition software (AcqKnowledge v.5.1, Biopac Systems) and low‐pass filtered (10 Hz edge frequency) offline.

### 2.7 Tendon morphological, mechanical and material properties

Details and reliability of the PT measurements have been documented elsewhere (Lee, Bridge, et al., 2023). Briefly, PT CSA was measured at 25%, 50% and 75% tendon length using a 4‐cm‐wide 5–18 MHz linear probe (Philips EPIQ 7 Ultrasound System, Bothel, USA) while participants were seated on the isokinetic dynamometer in the resting state, with the knee secured at 90°. Images of CSA were measured offline using ImageJ software (ImageJ v.1.8.0, National Institutes of Health, USA). After measuring tendon CSA and isometric MVCs, participants performed an isometric KE ramped maximal voluntary contraction (RMVC), which lasted 6 s. During the RMVC, a 10‐cm‐wide (10–15 MHz) linear probe (Mylab70, Esaote Biomedica, Italy) was positioned sagittally over the tendon to record tendon elongation. At least two RMVC attempts were made with 2 min rest in between attempts. Given that the loading rate (in newton metres per second) depended on the ability of the participant to produce maximal voluntary force, real‐time torque–time data were projected in front of the participants, in order that they could gradually and consistently increase torque output to MVC. Patellar tendon force was estimated by dividing KE torque during the RMVC by the tendon moment arm, which was estimated from the femur length of the participant (Visser et al., 1990).

The PT force was subsequently corrected for antagonist (hamstring) co‐activation via EMG. Using semi‐automated tracking software (Tracker, v.6.1.2, https://physlets.org/tracker/), the displacement of both the patellar apex and the tibial tuberosity was measured during RMVC. Individual tendon force–elongation data were subsequently fitted with a second‐order polynomial (*R* ^2 ^> 0.93 in all cases). Patellar tendon mechanical and material properties pre‐ and post‐training were calculated using the weakest maximum tendon force for each participant, which was determined during the pretraining tests. Patellar tendon strain was defined as tendon elongation expressed as a percentage of the original length of the tendon, i.e., 100 × change in tendon length (Δ*L*)/resting tendon length (*L*_0_). Tendon stress was defined as the peak tendon force (*F*_t_) at KE RMVC relative to the mean tendon CSA (i.e., *F*_t_/CSA). Tendon stiffness (Δ*F*_t_/Δ*L*) was calculated from the highest 20% *F*_t_ interval produced by the participant. Young's modulus (*E*) was calculated by multiplying stiffness (*k*) by the ratio of the resting tendon length to mean tendon CSA, i.e., *E* = *k*(*L*_0_/CSA).

### 2.8 Thickness of subcutaneous adipose tissue and skeletal muscle

The vastus lateralis (VL) muscle and its overlying subcutaneous adipose tissue (SAT) thickness were measured with the centre of the 10‐cm‐wide (10−15 MHz) linear probe (Mylab70, Esaote Biomedica, Italy) placed at 50% VL length and 50% VL width, while the participant sat on the isokinetic dynamometer in the resting state (with knee and hip angles as stated above). The thickness of both the VL and its SAT were an average of three measurements made for each tissue, i.e., at 25%, 50% and 75% of the 10‐cm‐wide field of view in the ultrasound image. Tissue thickness was measured offline using ImageJ software (ImageJ v.1.8.0, National Institutes of Health, USA).

### 2.9 Statistical analyses

All data are presented as mean values ± SD. Pretraining between‐group (COL vs. PLA) comparisons of physical characteristics and dietary intake were performed with Student's unpaired *t*‐tests. Two‐way mixed ANOVA models (group: COL vs. PLA; time: pre‐ vs. post‐training) were performed to detect changes in KE and KF MVC torque, resting PT length, loading rate, mean PT CSA, VL and its SAT thickness, and all other PT mechanical and material properties. When significant group × time interaction effects were found, *post hoc* Student's paired *t*‐tests (pre‐ vs. post‐training for COL and PLA) and Student's unpaired *t*‐tests (COL vs. PLA for percentage changes in tendon properties) were performed to reveal within‐ and between‐group differences over time. For PT CSA, a three‐way mixed ANOVA was performed to assess differences among group (COL vs. PLA), time (pre‐ vs. post‐training) and location (25% vs. 50% vs. 75% tendon length). Two effect sizes, Cohen's *d* (for *t*‐tests) and the partial eta squared (η_p_^2^; for ANOVA interaction) were reported for each statistical model. The thresholds of Cohen's *d* and η_p_^2^ are defined as small (*d *= 0.20 and η_p_^2^ = 0.01), medium (*d *= 0.50 and η_p_^2^ = 0.06) and large (*d *= 0.80 and η_p_^2^ = 0.14) (Cohen, 1988). Data were analysed by using the statistical software package SPSS (v.26, SPSS, Chicago, IL, USA), and the level of significance was set at *P *< 0.05.

## 3 RESULTS

### 3.1 Group characteristics

Age, body mass, height and baseline isometric KE did not differ between COL and PLA groups (all *P* > 0.05).

### 3.2 Macro‐ and micronutrient intake

Habitual and total nutritional intake is reported in Table 1. Energy, macronutrient and vitamin C intake did not differ between COL and PLA groups (all *P *> 0.05; Table 1).

### 3.3 Maximum strength and muscle thickness

Isometric and concentric KE MVC, isometric KF MVC and antagonist co‐activation before and after training are presented in Table 2. There were no main effects of training, group or interaction effects for any of the variables (*P *> 0.05). There were also no main effects of training, group or interaction effects for VL and SAT thickness (*P *> 0.05).

**TABLE 2:** Knee extension (KE) and knee flexion (KF) isometric and concentric maximal voluntary contraction (MVC) torque, antagonist muscle co‐activation and vastus lateralis (VL) muscle and subcutaneous adipose tissue (SAT) thickness in COL and PLA groups before (PRE) and after (POST) training. Data are means ± SD.

	COL (*n* = 6)		PLA (*n* = 5)		
Variable	PRE	POST	PRE	POST	Group × time, *P*‐value
Isometric exercise					
KE (N m)	190 ± 32	196 ± 47	204 ± 37	213 ± 27	0.850
KF (N m)	82.0 ± 15.7	85.1 ± 19.2	87.0 ± 20.5	89.6 ± 21.0	0.932
Concentric exercise					
KE (N m)	147 ± 26	140 ± 24	159 ± 22	162 ± 28	0.191
Antagonist co‐activation (%)	17.3 ± 5.9	16.7 ± 6.6	21.8 ± 7.4	16.8 ± 8.0	0.136
SAT thickness (mm)	8.0 ± 3.1	8.2 ± 3.1	8.4 ± 3.0	8.2 ± 3.0	0.611
VL thickness (mm)	27.0 ± 2.6	27.1 ± 2.7	26.9 ± 2.9	26.8 ± 2.8	0.163

### 3.4 Morphological, mechanical and material tendon properties

#### 3.4.1 Resting tendon CSA and length

Regarding the mean tendon CSA, there was a main effect of training (*F*_1,9_ = 6.914, *P* = 0.027, η_p_^2^ = 0.434) but no main effect for group (*F*_1,9_ = 0.249, *P *= 0.630, η_p_^2^ = 0.07) and no training × group interaction (*F*_1,9_ = 0.007, *P *= 0.934, η_p_^2^ = 0.001; Table 3). A three‐way ANOVA for tendon CSA at different locations revealed only a main effect of training (*F*_1,9_ = 6.910, *P* = 0.027, η_p_^2^ = 0.434), i.e., no regional hypertrophy in either group. There were no main effects for training or group and no interaction effects for resting tendon length (*P *> 0.05; Table 3).

**TABLE 3:** Patellar tendon morphological, mechanical and material properties in PLA and COL groups before (PRE) and after (POST) training. Data are means ± SD.

	COL (*n* = 6)		PLA (*n* = 5)		
Variable	PRE	POST	PRE	POST	Group × time, *P*‐value
Resting tendon length (mm)	47.2 ± 5.6	47.3 ± 5.0	49.3 ± 4.1	49.3 ± 4.3	0.844
Mean CSA (mm^2^)	73.9 ± 5.7	74.7 ± 5.4	72.1 ± 6.1	72.9 ± 5.9	0.934
Tendon force (N)	4709 ± 754	4959 ± 1012	4323 ± 752	4721 ± 717	0.895
Stress (MPa)	59.7 ± 9.9	59.0 ± 10.0	55.3 ± 8.2	56.0 ± 7.8	0.145
Elongation (mm)	4.0 ± 0.4	3.9 ± 0.8	3.8 ± 0.9	3.5 ± 0.7	0.414
Strain (%)	8.5 ± 1.0	8.2 ± 1.5	7.8 ± 2.0	7.1 ± 1.6	0.508

#### 3.4.2 Tendon mechanical and material properties

**FIGURE 1:**
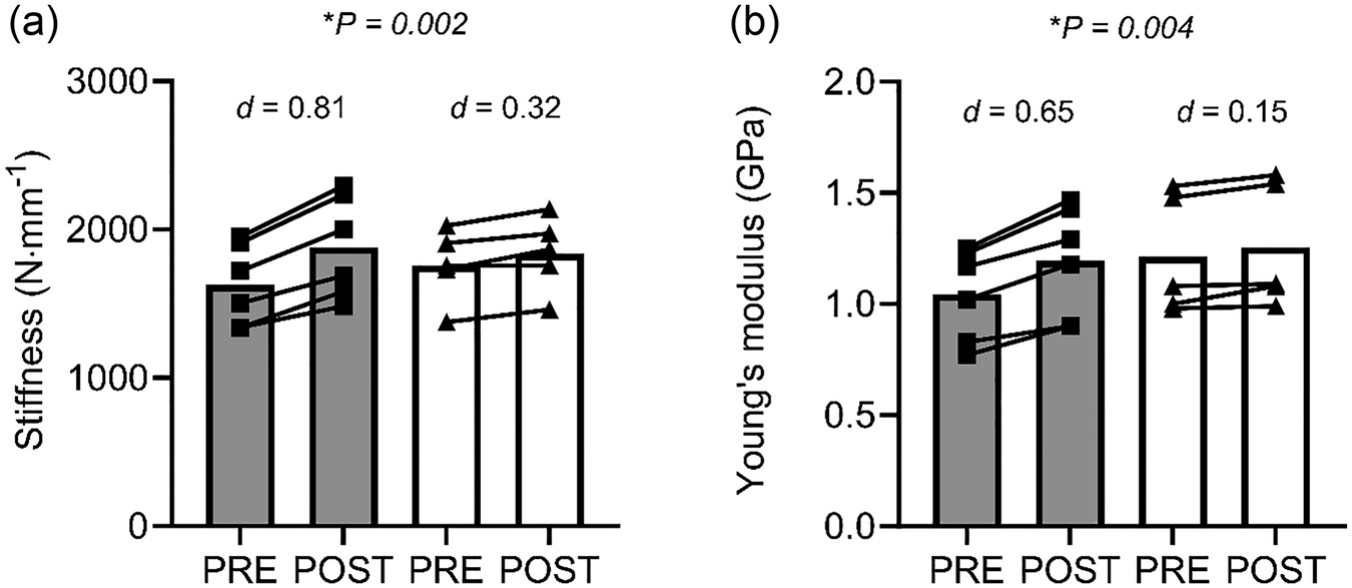
Tendon stiffness (a) and Young's modulus (b) before (PRE) and after (POST) the 10 weeks of soccer training in the collagen group (grey bar and black squares) and the placebo group (white bar and black triangles). *Group × time interaction effects. Abbreviation: *d*, Cohen's *d* effect size.

Concerning tendon stiffness, there was a main effect of training (*F*_1,9_ = 65.348, *P* < 0.001, η_p_^2^ = 0.879) and an interaction effect (*F*_1,9_ = 18.968, *P* = 0.002, η_p_^2^ = 0.666) but no main effect of group (*F*_1,9_ = 0.066, *P* = 0.804, η_p_^2^ = 0.007; Figure 1a). Regarding Young's modulus, there was a main effect of training (*F*_1,9_ = 47.424, *P* < 0.001, η_p_^2^ = 0.840) and an interaction effect (*F*_1,9_ = 15.005, *P* = 0.004, η_p_^2^ = 0.625) but no main effect of group (*F*_1,9_ = 0.578, *P *= 0.467, η_p_^2^ = 0.060; Figure 1b). Concerning the other tendon properties (tendon stress, strain and elongation), there were no main effects of training or group and no interaction effects (all *P *> 0.05; Table 3). *Post hoc* Student's paired *t*‐tests revealed that tendon stiffness increased significantly after 10 weeks of soccer training in both COL [*t*(5) = −7.865, *P *< 0.001] and PLA [*t*(4) = −3.471, *P *< 0.026] groups. Also, Young's modulus increased significantly in both COL [*t*(5) = −6.664, *P *= 0.001] and PLA (*t*(4) = 0.020, *P *= 0.039) groups. Consequently, *post hoc* Student's unpaired *t*‐tests revealed that percentage changes in tendon stiffness [*t*(9) = 5.891, *P *< 0.001, +15.4% ± 3.1% vs. +4.6% ± 3.0%] and Young's modulus [*t*(9) = 4.910, *P *< 0.001, +14.2% ± 4.0% vs. +3.4% ± 2.8%] were higher in COL vs. PLA. *Post hoc* power analyses with G*Power software (v.3.1.9.6), using the effect sizes from the time (pre and post 10 weeks of soccer training) × group (COL vs. PLA) interaction for the change in patellar tendon stiffness (η_p_^2^ = 0.666) and Young's modulus (η_p_^2^ = 0.625) revealed that the statistical power for the PT stiffness and Young's modulus group × time interactions were 96% and 93%, respectively (two‐way mixed ANOVA; α = 0.05). Loading rate during the 6 s RMVC was COL, 29.6 ± 5.3 N m s^−1^ versus PLA, 29.4 ± 5.5 N m s^−1^ (pretraining) and COL, 32.6 ± 7.2 N m s^−1^ versus PLA, 33.7 ± 7.9 N m s^−1^ (post‐training). There was a main effect of training (*F*_1,9_ = 11.993, *P* = 0.007, η_p_^2^ = 0.571) but no main effect of group (*F*_1,9_ = 0.011, *P* = 0.919, η_p_^2^ = 0.001) and no interaction effect (*F*_1,9_ = 0.387, *P* = 0.549, η_p_^2^ = 0.041). Therefore, any group differences in changes in mechanical and material tendon properties were not affected by loading rate in the present study.

## 4 DISCUSSION

The main aim of this study was to investigate the effect of collagen hydrolysate (COL) supplementation on the changes in PT morphological, mechanical and material properties after 10 weeks of soccer training during the pre‐season period (incorporating high‐intensity lower‐limb resistance and plyometric training) in professional female soccer athletes. The main findings were greater increases in tendon stiffness and Young's modulus in COL compared with PLA. Although there was a main effect of training on the change in PT CSA, the degree of tendon hypertrophy did not differ between COL and PLA.

We observed large effect sizes for changes in PT stiffness (*d *= 0.81 vs. *d *= 0.32) and moderate effect sizes for changes in Young's modulus (*d *= 0.65 vs. *d *= 0.15) in COL compared with small effects sizes in PLA. Likewise, 10 weeks of in‐season soccer training incorporating lower‐limb bodyweight resistance/plyometric exercise supplemented with 30 g COL increased tendon stiffness and Young's modulus more than PLA in a women's soccer academy squad, although no patellar tendon hypertrophy was observed in either group (Lee, Bridge, et al., 2023). The exercise intensity used in the study by Lee, Bridge, et al. (2023) was probably insufficient to induce patellar tendon hypertrophy at the proximal and distal ends of the patellar tendon (Dalgaard et al., 2019; Kongsgaard et al., 2007; Seynnes et al., 2009). Therefore, this study implemented high‐intensity (75%–90% one‐repetition maximum) lower‐limb RT once a week (in addition to loaded plyometric training once a week and regular pitch‐based training and matches), with the intention of inducing tendon hypertrophy. Indeed, there was a main effect of training, but COL did not show an augmented hypertrophic adaptation, and the effect sizes for the changes in tendon CSA for both COL and PLA were small (*d *= 0.1). Jerger et al. (2023, 2022) found that 14 weeks of RT (three times per week at 70%–85% one‐repetition maximum) with 5 g daily hydrolysed collagen intake increased Achilles and PT CSA more than PLA in young healthy men (Jerger et al., 2023, 2022). Therefore, trivial changes in PT CSA in the present study might be attributable to the relatively low frequency of high‐intensity RT. Alternatively, the fact that these athletes were performing RT on a regular basis prior to the study means that the musculoskeletal system of the athletes had probably adapted to this intensity and frequency of RE, which would also explain the lack of strength gains and muscle hypertrophy observed in both groups after the 10‐week intervention. Furthermore, during pitch‐based sessions (e.g., training and matches), soccer players run intermittently at different speeds and perform explosive movements (i.e., vertical jumps and change of direction) that increase tendon loading. Previous studies have found that human PT CSA increases with habitual loading (i.e., running or rapid forward lunge) (Couppé et al., 2008, 2014), which might also explain the limited tendon hypertrophy observed in our study. However, given the recent findings that 30 g COL ingested prior to RE increased whole‐body collagen synthesis more than 15 and 0 g COL (Lee, Tang, et al., 2023), the greater changes in PT stiffness and Young's modulus in the COL versus PLA group in the present study were probably attributable to augmented mechanical loading‐induced tendon collagen synthesis (Lee, Tang, et al., 2023; Miller et al., 2005) in the presence of high serum concentrations of the necessary exogenous amino acids (i.e., glycine and proline) for synthesizing new collagen (Lee, Tang, et al., 2023b, 2025).

Despite the main effect of time and group × time interaction effect for both PT stiffness and Young's modulus, there was no main effect of time or group × time interaction effect for maximal strength or VL muscle thickness. This was not unexpected, owing to the relatively low collagen content in skeletal muscle (∼7%) compared with the much greater content (∼85%) in tendon (Babraj et al., 2005; Kjaer, 2004) and the relatively low muscle protein synthesis response by skeletal muscle to hydrolysed collagen ingestion following RE (Oikawa et al., 2020). Moreover, our participants were professional athletes, who regularly participated in RT as part of their habitual training, hence their neuromuscular system probably had less capacity to adapt. Tendon stiffness and modulus, in contrast, are highly responsive to changes in (de)training (Couppé et al., 2012); therefore, despite the trained status of our participants, we expected there to be some detraining effect on the tendon following the ∼8‐week ‘off‐season’ period (prior to the start of our study, which commenced at the start of the ‘pre‐season’ period). This would have allowed the tendon to demonstrate significant changes in stiffness and modulus, albeit to a lesser extent to those seen after similar length RT interventions in previously untrained participants (Seynnes et al., 2009).

We acknowledge that the small sample size in this study (owing to the ∼40% participant withdrawal) was a limitation. However, professional athletes have restricted availability for participating in research and are at risk of sustaining injuries related to their sport, meaning that participant withdrawal from research studies involving elite/professional athletes is not unusual. Furthermore, some of our participants became infected with COVID‐19 during the course of the study, which took place during the COVID‐19 pandemic, and also had to withdraw. Although our *post hoc* power analyses for the changes in PT stiffness and Young's modulus demonstrated 96% and 93% power, respectively (suggesting that the study was powered to detect these changes), the large effect sizes used to calculate statistical power might have been inflated owing to the limited sample size (Button et al., 2013). However, our results are in line with those reported in larger samples of female academy soccer players (Lee, Bridge, et al., 2023) and previously untrained young men (Jerger et al., 2023) regarding PT adaptations to RT and hydrolysed collagen supplementation, thus providing confidence in our findings. Furthermore, owing to the limited availability of the professional athletes who participated in our study, we were unable to assess the muscle–tendon properties of participants during the same menstrual cycle phase in the pre‐ and post‐training tests. We also decided not to exclude volunteers based on use of hormonal contraceptives, and we acknowledge that these factors might have affected our findings. However, our study design reflects the real‐world environment of women's professional sport, in that athletes must be able to perform at any point during their menstrual cycle, and our results should be generalizable to all professional female soccer athletes, regardless of menstrual cycle phase or hormonal contraceptive use.

Given that a stiffer tendon can tolerate greater stress (LaCroix et al., 2013) and transmit muscle force more efficiently (Bojsen‐Møller et al., 2005), the practical implications of our findings include the potential mitigation of tendon injury risk and improvement in physical performance involving rapid force production. Furthermore, given the positive effects of overloading on other connective tissues, such as ligament (Kharaz et al., 2021) and bone (Beyer et al., 1985), and the similar collagen composition of tendon and ligament (Kjaer et al., 2015), it is feasible that such benefits might not be limited only to tendon. However, future studies would need to test this hypothesis with specific measurements not included in this study, in addition to an RT programme designed specifically to target those tissues.

In conclusion, 10 weeks of pre‐season soccer training (incorporating high‐intensity lower‐limb RT), supplemented with 30 g collagen and 500 mg vitamin C three times a week, conferred greater gains in patellar tendon stiffness and Young's modulus in comparison to soccer training alone in professional female soccer athletes.

## AUTHOR CONTRIBUTIONS

Research concept and study design (Robert M. Erskine); data collection (Joonsung Lee, David C. Robshaw); data analysis (Joonsung Lee, Robert M. Erskine); writing—original draft (Joonsung Lee); writing—review, editing and approval of final draft (Robert M. Erskine, David C. Robshaw). All authors approved the final version of the manuscript and agree to be accountable for all aspects of the work in ensuring that questions related to the accuracy or integrity of any part of the work are appropriately investigated and resolved. All persons designated as authors qualify for authorship, and all those who qualify for authorship are listed.

## CONFLICT OF INTEREST

None declared.

## FUNDING INFORMATION

None.

## Supporting information


**Table S1**. The externally loaded lower‐limb resistance and plyometric exercises.

**Table S2**. The number of nutritional supplements participants had with training sessions or match. Data are means ± SD.

**Figure S1**. A CONSORT flow diagram showing participant recruitment and intervention time line. Abbreviations: COL, collagen group; PLA, placebo group.

## Data Availability

Data described in the article will be made available upon request from the corresponding author pending application and approval.
