# Supplementary material for: High‐intensity resistance training and collagen supplementation improve patellar tendon adaptations in professional female soccer athletes
Source: Exp Physiol. 2024 Aug 29;110(11):1551–60. doi: 10.1113/EP092106 (PMC12576002; doi:10.1113/EP092106)
Supplement: Supplementary file 1 — Table S1. The externally loaded lower‐limb resistance and plyometric exercises. [file EPH-110-1551-s002.docx]

**Supplementary Table 1.** The externally loaded lower-limb resistance and plyometric exercises.

| Lower-limb resistance exercise | | | | |
| --- | --- | --- | --- | --- |
| Weeks 1 – 8 | | | | |
| Exercise | Volume and intensity | Week 1 | Week 2 | Week 3 – 8 |
| Bilateral ballistic exercise |  |  |  |  |
| Clean pull from rack or  loaded jump or trap bar pulls or  medicine ball countermovement  jump | Set | 2 | 3 | 4 |
|  | Repetitions | 4 | 4 | 4 |
|  |  |  |  |  |
| Unilateral anterior exercise |  |  |  |  |
| Rear-foot elevated split squat or  split squat | Set | 3 – 4 | | |
|  | Repetitions | 2 – 6 | | |
|  | Intensity | 75 – 90 % 1RM | | |
|  |  |  | | |
| Hip dominant posterior chain exercise  Staggered Romanian deadlift or  hip thrust |  |  |  |  |
|  | Set  Repetitions | 2  5 | 3  5 | 4  5 |
|  |  |  |  |  |
| Unilateral plantar flexor exercise  Single-leg calf raise or  calf-raise isometric hold (5- or 10-s  hold) |  |  |  |  |
|  | Set  Repetitions | 2  8 | 3  8 | 3  6 |
|  |  |  |  |  |
|  |  |  |  |  |
| Copenhagen adduction exercise | Set | 2 | 2 | 2 – 3 |
|  | Repetitions | 6 | 8 | 8 – 15 |
|  |  |  |  |  |
|  |  |  |  |  |
| Nordic hamstring exercise | Set | 1 | 2 | 3 |
|  | Repetitions | 3 | 3 | 3 |
|  | | | | |
| Week 9 – 10 | | | | |
| Exercise | Volume and intensity | Week 9 – 10 | | |
| Bilateral ballistic exercise  Hang high pull or  loaded jump or  trap bar pulls or  medicine ball countermovement  jump |  |  | | |
|  | Set  Repetitions | 2 – 3  4 – 6 | | |
|  |  |  | | |
| Unilateral anterior exercise |  |  | | |
| Rear-foot elevated split squat | Set | 3 | | |
|  | Repetitions | 4 | | |
|  | Intensity | 75 – 90% 1RM | | |
|  |  |  | | |
| Hip dominant posterior chain exercise  Romanian deadlift or  single-leg glute-hamstring raise |  |  | | |
|  | Set  Repetitions | 2 – 4  4 – 6 | | |
|  |  |  | | |
| Unilateral plantar flexor exercise  Single-leg calf raise or  calf-raise isometric hold (5 s hold) |  |  | | |
|  | Set  Repetitions | 2 – 3  3 | | |
|  |  |  | | |
| Drop jump to box | Set | 2 – 3 | | |
|  | Repetitions | 3 | | |
|  |  |  | | |
| Nordic hamstring exercise | Set | 2 – 3 | | |
|  | Repetitions | 3 | | |
|  |  |  | | |
| Plyometric exercise | | | | |
| Week 1 – 10 | | | | |
| Broad jump | Set  Repetitions | 2 – 4  5 | | |
| Seated box jump | Set  Repetitions | 2 – 4  5 | | |
| Pogo jumps | Set  Repetitions | 2 – 4  10 | | |

1RM, one-repetition maximum.
