# Supplementary material for: High‐intensity resistance training and collagen supplementation improve patellar tendon adaptations in professional female soccer athletes
Source: Exp Physiol. 2024 Aug 29;110(11):1551–60. doi: 10.1113/EP092106 (PMC12576002; doi:10.1113/EP092106)
Supplement: Supplementary file 2 — Table S2. The number of nutritional supplements participants had with training sessions or match. Data are means ± SD. [file EPH-110-1551-s003.docx]

**Supplementary Table 2.** The number of nutritional supplements participants had with training sessions or match. Data are mean ± SD.

| **Session type** | **COL (*n* = 6)** | **PLA (*n* = 5)** |
| --- | --- | --- |
| PBS | 13 ± 1 | 13 ± 1 |
| PBS and RE | 7 ± 1 | 8 ± 1 |
| PBS and PLY | 10 ± 0 | 9 ± 1 |
| Match | 0 ± 0 | 0 ± 0 |

*PBS,* pitch-based session; *PLY,* plyometric exercise; *RE,* resistance exercise.
