# Supplementary material for: High‐intensity resistance training and collagen supplementation improve patellar tendon adaptations in professional female soccer athletes
Source: Exp Physiol. 2024 Aug 29;110(11):1551–60. doi: 10.1113/EP092106 (PMC12576002; doi:10.1113/EP092106)
Supplement: Supplementary file 3 — Figure S1. A CONSORT flow diagram showing participant recruitment and intervention time line. Abbreviations: COL, collagen group; PLA, placebo group. [file EPH-110-1551-s001.pdf]

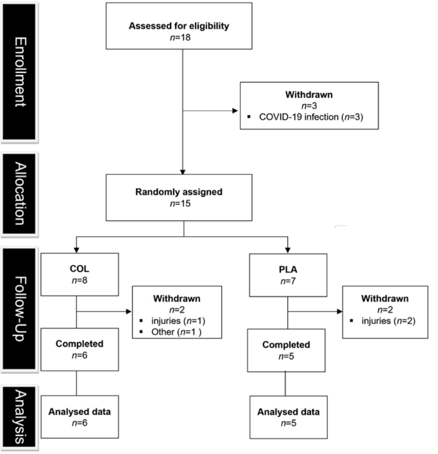
Enrollment
Allocation
Follow-Up
Analysis
Assessed for eligibility
n=18
Withdrawn
n=3
COVID-19 infection (n=3)
Randomly assigned
n=15
COL
n=8
PLA
n=7
Withdrawn
n=2
injuries (n=1)
Other (n=1)
Withdrawn
n=2
injuries (n=2)
Completed
n=6
Completed
n=5
Analysed data
n=6
Analysed data
n=5
